# Understanding Kratom Use: A Guide for Healthcare Providers

**DOI:** 10.3389/fphar.2022.801855

**Published:** 2022-03-02

**Authors:** Marc T. Swogger, Kirsten E. Smith, Albert Garcia-Romeu, Oliver Grundmann, Charles A. Veltri, Jack E. Henningfield, Lorna Y. Busch

**Affiliations:** ^1^ Department of Psychiatry, University of Rochester Medical Center, Rochester, NY, United States; ^2^ Translational Addiction Medicine Branch, National Institute on Drug Abuse Intramural Research Program, Baltimore, MD, United States; ^3^ Department of Psychiatry and Behavioral Sciences, Johns Hopkins University School of Medicine, Baltimore, MD, United States; ^4^ Department of Pharmaceutical Sciences, Midwestern University College of Pharmacy, Glendale, AZ, United States; ^5^ College of Pharmacy, Department of Medicinal Chemistry, University of Florida, Gainesville, FL, United States; ^6^ Pinney Associates, Bethesda, MD, United States

**Keywords:** kratom (*Mitragyna speciosa* Korth), emerging therapeutic agents, pain, mood and anxiety, substance use and misuse

## Abstract

Kratom (*Mitragyna speciosa* Korth., Rubiaceae) is a plant native to Southeast Asia, where it has been used for centuries as a mild stimulant and as medicine for various ailments. More recently, as kratom has gained popularity in the West, United States federal agencies have raised concerns over its safety leading to criminalization in some states and cities. Some of these safety concerns have echoed across media and broad-based health websites and, in the absence of clinical trials to test kratom’s efficacy and safety, considerable confusion has arisen among healthcare providers. There is, however, a growing literature of peer-reviewed science that can inform healthcare providers so that they are better equipped to discuss kratom use with consumers and people considering kratom use within the context of their overall health and safety, while recognizing that neither kratom nor any of its constituent substances or metabolites have been approved as safe and effective for any disease. An especially important gap in safety-related science is the use of kratom in combination with physiologically active substances and medicines. With these caveats in mind we provide a comprehensive overview of the available science on kratom that has the potential to i clarity for healthcare providers and patients. We conclude by making recommendations for best practices in working with people who use kratom.

## Introduction

Kratom (*Mitragyna speciosa* Korth., Rubiaceae; also known as ketum) is made from the leaves of a tropical tree in the coffee family indigenous to Southeast (SE) Asia, where it has been used for centuries as medicine for various ailments, including hypertension, diarrhea, cough, and fever (Tanguay, 2011; Cinosi et al., 2015; Singh et al., 2016). Despite such traditional medicinal use, it is important to recognize that neither kratom, nor its constituents (e.g., “alkaloids”), nor metabolites have been approved as safe and effective medicines for any therapeutic use. Nonetheless, widespread use for health and well-being include diverse uses reported by consumers as reasons for their use. For example, at low doses, kratom has long been consumed orally as a stimulant to enhance stamina and productivity, making it particularly popular among field laborers working long days in arduous conditions (Tanguay, 2011; Prozialeck et al., 2012; Hassan et al., 2013; Warner et al., 2016). Consumption remains widespread in kratom’s native lands, where people commonly chew raw kratom leaves or boil leaves to make tea (Swogger and Walsh, 2018). Kratom can also be smoked, vaporized, or consumed as a powder. Because of its purported analgesic properties, kratom is used to treat pain and, notably, as a means to alleviate opioid withdrawal or as an opioid replacement among people with opioid use disorder (OUD) (Smith and Lawson, 2017; Henningfield et al., 2018; Bath et al., 2020). In addition to analgesia produced at higher doses, kratom is reported to have relaxing, anxiolytic effects. Over the past 2 decades, kratom has gained popularity beyond Asian borders, particularly in North America and Europe (Boyer et al., 2007; Grundmann, 2017).

An estimated 10–16 million people in the United States take kratom, though current prevalence ranges of 1.3%–6.1% from national representative surveys may underestimate regular kratom users (Henningfield et al., 2019; Covvey et al., 2020). Whereas in Southeast Asia users typically buy kratom leaves directly from a grower, Westerners often purchase capsules, powders, or extracts via the internet, specialty smoke shops, and gas stations (Prozialeck et al., 2012; Singh et al., 2016)*.* Kratom is currently not recognized as a dietary supplement in the United States, and the Food and Drug Administration (FDA) has not issued guidance or regulatory standards on kratom regarding allowable product contents, alkaloid concentrations, packaging, labeling, or marketing of kratom products that is usually provided for dietary ingredients (Coe et al., 2019). This gap in regulatory policy prompted the American Kratom Association (AKA) to develop voluntary industry guidelines through a Good Manufacturing Practice (GMP) Standards Program that tests for purity and contaminants (American Kratom Association, 2019). Due to the potential for adulteration of kratom products the unregulated status of kratom in most United States remains a concern.

Although the rise of kratom use in the West has been an opportunity for increased scientific study, the resultant publication of a great deal of research of limited rigor has created confusion for health practitioners attempting to understand the benefits and risks of the plant and the heterogeneity of kratom products. Case studies, poison control center briefings, and tallied coroner and medical examiners’ reports have disproportionately emphasized, as these forms of inquiry often do, extreme and rare events, including seizure, liver damage, and death (e.g., Nelsen et al., 2010; Sheleg and Collins, 2011; Kapp et al., 2011; Neerman et al., 2013; Anwar et al., 2016; Wang and Walker, 2018; Post et al., 2019; Afzal et al., 2020), even as some have elucidated that adverse health outcomes from kratom exposure have been mild to moderate and resolved quickly (Anwar et al., 2016). Still, there remains considerable ambiguity on the potential harms from kratom use. In February 2018, the FDA cited 44 cases of kratom-associated deaths based upon coroner or forensic toxicologist reports. However, at the current level of scientific knowledge, several factors make it *impossible* to determine whether kratom contributed to lethal outcomes. Almost all of the cases cited involved adulterated kratom products and/or the co-ingestion of substances with fatal overdose potential, including heroin and synthetic opioids (Babin, 2018). For instance, nine deaths were from an herbal mix, Krypton, containing a metabolite of the opioid tramadol (Bäckstrom et al., 2010). Additionally, the mere presence of mitragynine (one of kratom’s primary alkaloids believed to be responsible for analgesia) in decedents’ plasma or evidence of presumed kratom consumption (e.g., kratom product packages) does not implicate the plant’s role in toxicity, especially given the large variability of mitragynine serum levels of decedents, ranging from 5.6 to 29,000 ng/ml (Papsun et al., 2019). Finally, there is no clear mechanism by which kratom alone and taken even at high doses would directly cause death. Unlike classical opioids, which act as full agonists at mu opioid receptors, kratom’s two primary and best understood bioactive alkaloids, mitragynine and 7-hydroxymitragynine, act at mu opioid receptors as partial putatively “biased” agonists, meaning that they do not contribute to significant respiratory depression in pre-clinical animal studies (as discussed more below), making “poisoning”, when kratom alone is used, a highly questionable cause of death. Importantly, there are no reports of deaths due to kratom use in SE Asia for over a century (Veltri and Grundmann, 2019). Despite insufficient evidence for kratom’s role in harm, media headlines misleadingly insinuate that kratom has been established as a cause of death (e.g., “kratom deaths” and “kratom overdose deaths; ” Galvin, 2019; Kaur, 2019; Miller, 2019).

Conclusions by many negative, sensationalized, or otherwise decontextualized media reports on kratom have been questionably drawn from case studies and toxicology reports which, at best, provide low levels of evidence due to unknown internal validity and generalizability and over-representation of extreme events (Merriam, 2009). Unfortunately, warnings regarding kratom exhibit features of *drug hysteria* (Hart, 2013), which involves the promulgation of sensational and biased information and the pursuit of legislative approaches that are disproportionate to apparent public health risks. At the public health level, drug hysteria is not only scientifically unfounded, but dangerous. In the case of kratom, misinformation can lead to dehumanization of kratom users, disinclination for people with OUD to try kratom as a substitute for opioids that are causing them harm, and the continued promotion of ineffective, draconian, and punitive policies with the potential to contribute to mass incarceration, a serious public health threat in its own right. Simultaneously, drug hysteria can contribute to the inhibition of rigorous scientific study and thereby deprive the public of scientifically-informed pharmacotherapeutic interventions (PR Newswire, 2016). Banning or criminalizing kratom, as six United States have done at the time of this writing, has the potential to create a new illicit market for kratom products, increasing the likelihood of adulteration and the use of dangerous substances as kratom substitutes. All of this results in harm to people who regularly use kratom to address pain, psychiatric problems, and SUD symptoms (Grundmann, 2017; Swogger and Walsh, 2018; Coe et al., 2019; Smith et al., 2021a; Smith et al., 2021b). Moreover, sensationalized and negative reports lead some patients to fear revealing kratom use to their healthcare providers (Smith et al., 2021b) and misinform those providers about the risks of kratom use.

Subsequent to increased kratom use in the United States, an eight-factor analysis (8 FA) normally required prior to scheduling decisions was performed by the FDA, and another by an independent agency (Pinney Associates). The former has been criticized by kratom researchers for omission of important scientific studies and pertinent data, as well as inappropriate use of a computer simulation model (PHASE) that provided data that the FDA used to deem kratom an “opioid” (Grundmann et al., 2018), linking it to more dangerous classical opioids without providing information on differences between kratom and these drugs. Meanwhile, Pinney Associates concluded that kratom is distinct from classical opioids and poses no more of a public health risk than many commonly-used substances, thereby warranting product oversight rather than a ban. Nonetheless, recent publications in medical journals espouse kratom use as “highly problematic” and its effects “contributors to the growing opioid crisis” (e.g., Goldin et al., 2019), without adequate supporting data. This rhetoric can stigmatize users and mislead well-intentioned healthcare professionals into an anti-kratom stance that could negatively impact their patients and the patient-provider relationship.

A balanced examination of what can be drawn from the existing literature directed to healthcare providers and clinicians is warranted. This is particularly true given that the study of kratom is in its infancy: there is only one published clinical trial of kratom’s effects in humans. There is, however, a growing body of observational literature that represents a higher level of evidence than case reports or forensic toxicologists’ and medical examiners’ reports. Here, we first review research on the pharmacology of kratom and then summarize the available observational science on human kratom use in order to provide the most nuanced, accurate, and comprehensive review of kratom’s potential benefits and risks possible at this early stage of kratom research. We acknowledge that information provided here will inevitably change as more data are collected on kratom and kratom use. Like all things in science, our understanding of this plant and its use is provisional. Here we provide the most up-to-date information in an accessible manner. Based on the review, we conclude with recommendations to health practitioners for conceptualizing kratom use and working with patients who use kratom.

## Pharmacology and Animal Studies

Of the dozens of alkaloids identified in kratom, mitragynine is the most prominent (comprising approximately 60 percent; Hassan et al., 2013) and appears, along with 7-hydroxymitragynine, to be primarily responsible for the plant’s unique psychoactive properties, which include opioid and non-opioid activities (Adkins et al., 2011; Kruegel and Grundmann, 2018; Raffa et al., 2018) that are dose dependent. In relatively low doses (<5 g), kratom has stimulant properties similar to its coffee relative, while larger quantities may produce sedating and analgesic effects (Kruegel and Grundmann, 2018; Coe et al., 2019; Kruegel et al., 2019; Todd et al., 2020). 7-hydroxymitragynine, while more potent than mitragynine, is unlikely to contribute to pharmacological effects due to its low natural presence in kratom leaves (Kruegel and Grundmann, 2018; Todd, et al., 2020; Obeng et al., 2021). Mitragynine is metabolized by humans via CYP enzymes into 7-hydroxymitragynine but the amount generated via metabolism is not sufficient to explain the analgesic effects of kratom products as a whole (Kamble et al., 2019; Maxwell et al., 2021).

*In vitro* studies reveal that biochemical pathways responsible for the analgesic and sedating effects of kratom do not carry risk of overdose comparable to classical opioids. Specifically, mitragynine and 7-hydroxymitragynine have partial affinity for the *mu* opioid receptor (Kapp et al., 2011; Prozialeck et al., 2012), whereas morphine is a full agonist. Binding of kratom alkaloids to this receptor largely activate G-protein coupled pathways, as opposed to the beta-arrestin pathway responsible for classical opioids’ common deadly side effect of respiratory depression (Kruegel et al., 2016; Váradi et al., 2016; White, 2018; Kruegel et al., 2019; Basiliere and Kerrigan, 2020; Behnood-Rod et al., 2020). Mitragynine also exerts non-opioid receptor pain-relieving effects by stimulating alpha-2 adrenoceptors and inhibiting cyclooxygenase-2 messenger RNA (mRNA) and protein expression (Matsumoto et al., 1996).

The distinct affinity for and activation of opioid receptors, as well as non-opioid analgesic effects that clearly distinguish kratom from classical opioids (Raffa et al., 2018), may explain why there are relatively few kratom-related safety issues given its widespread use. It is likewise important to keep in mind that while many effects of kratom are mediated by opioid receptors, kratom’s pharmacology indicates additional non-opioid mechanisms of action, including for mitragynine, again underscoring the complexity of the plant and our limited knowledge of its pharmacology (Hiranita et al., 2019). In addition to limitations in understanding the mechanisms of action and toxicity of kratom, is the limitation in data addressing use of kratom in combination with approved medicines, illicit drugs, and other herbal products. By way of example, the partial opioid agonist buprenorphine, which is approved by many regulatory agencies globally for the treatment of opioid withdrawal and use disorder, as well as pain, carries a far lower risk of lethal respiratory depression when used alone, but has been identified as contributing to overdose deaths when used in combination with benzodiazepines and other sedatives (Kumar et al., 2021). There has not been sufficient study to determine if kratom in combination with benzodiazepines and other sedatives, carries similar, greater, or lessor risks as compared to buprenorphine, so it would seem prudent for health care providers and kratom consumers to be aware of such limitations in the evidence and avoid such combinations and to minimize intake levels when combination consumption occurs because risks with most substances tend to be dose-related. The same cautions apply to use in combination with other substances.

The pharmacokinetics of mitragynine have been established in rodents, primarily rats, following oral administration (Ramachandram et al., 2019). Depending on the vehicle preparation, maximum plasma concentration, c_max_ (0.42–0.70 μg/ml), time to reach c_max_, t_max_ (1.26–4.50 h), and elimination half-life, t_1/2_, (3.85–9.43 h) indicated that mitragynine was highly variable in its absorption and/or metabolism. A study using traditionally prepared kratom tea and a hydroalcoholic kratom extract given orally to rats resulted in a c_max_ of 63.8 and 111.9 ng/ml and t_max_ of 1.3 and 3.1 h, respectively, while the t_1/2_ was not determined (Kamble et al., 2021). This supports the conclusion that the absorption of mitragynine is influenced by the presence of other kratom leaf compounds. Only one human study to date evaluated the pharmacokinetics of mitragynine following oral administration of a traditionally prepared kratom tea in 10 male volunteers (Trakulsrichai et al., 2015). The pharmacokinetic parameters for mitragynine were an average t_max_ of 0.83 h, c_max_ ranging from 0.0185 to 0.105 μg/ml, and an average terminal t_1/2_ of 23.2 h. The maximum plasma concentration depends largely on the dose administered and thus needs to be interpreted within that context. However, both the time to reach maximum plasma concentration and half-life are usually comparable, at least within the same species. Because there is only one human study reporting mitragynine pharmacokinetics and substantial variability was found in rat studies, it is too early to conclude how well animal data can predict mitragynine pharmacokinetics in humans. Furthermore, the kratom preparation may impact the absorption and pre-systemic metabolism of mitragynine and other active principles.

Animal research provides further evidence of kratom’s relative safety compared to classical opioids. Studies aimed to establish lethal kratom doses have not induced any acute deaths with symptoms similar to morphine. Instead, at doses of mitragynine equivalent to hundreds or more times the typical human dose range, some animals died within days or weeks from a variety of causes unrelated to respiratory depression (Henningfield et al., 2018; Prozialeck et al., 2019; Henningfield et al., 2022). Kratom doses of up to 807 mg/kg in rats or 920 mg/kg in dogs did not indicate signs of toxicity (Macko et al., 1972). Animal studies evaluating reinforcing effects through intravenous self-administration reveal that, unlike morphine, mitragynine does not serve as a reinforcer in rats (Hemby et al., 2018; Yue et al., 2018) and therefore has lower abuse potential. Mitragynine was also found to reduce rodent morphine (Hemby et al., 2018) and heroin self-administration (Yue et al., 2018). See Henningfield et al. (2022) in this special issue for an update of many more studies related to the abuse potential of kratom. Furthermore, administration of 7-hydroxymitragynine takes the equivalent of 100 times more than what humans consume to display reinforcing effects (Hemby et al., 2018). Rodent studies also demonstrate that prodigious amounts of mitragynine (not ingestible at a human equivalent) may be needed to produce severe and sustained withdrawal effects that rival those produced by classical opioids (Harun et al., 2015; Henningfield et al., 2018). Rather, kratom has been found to attenuate opioid withdrawal symptoms in animals, albeit with its own milder withdrawal effects after cessation of long-term use (Hassan et al., 2013; Sabetghadam et al., 2013; Yusoff et al., 2016). Rodent studies confirm physical withdrawal from kratom that occurs after injection with the opioid inhibitor naloxone (e.g., Matsumoto et al., 2005), as well as with cessation of repeated mitragynine administration (Yusoff et al., 2016). Symptoms include somatic withdrawal within 12 h and increased anxiety, evident after 24 h. Across studies, dose-dependent indicators of both toxicity and withdrawal related to isolated kratom alkaloids have been found to resolve after discontinuation or a short duration of time has passed, respectively.

## Observational Research

Our current understanding of kratom’s effects in humans are based primarily on observational studies, including those using surveys, online experience reports, and/or validated self-report measures. With the increasing popularity of kratom in the West, online surveys (assessing tens of thousands of kratom consumers) have been conducted by United States researchers revealing that unlike SE Asia, where kratom appears to be predominantly consumed by males, almost half of United States consumers are female. Also, a majority of Western consumers are middle-aged, middle-income, Caucasian, and college-educated with private insurance. Most discovered kratom through the Internet or social media, about 25% from an acquaintance/friend, and a mere 3% from a healthcare provider. Only approximately 40% informed their healthcare providers about their use (Grundmann, 2017). Generally, motives for use in the West mirror those in SE Asia and include improvements in health, well-being, and productivity. Survey respondents overwhelmingly indicate that regular kratom consumption produces desired effects, including relief of various symptoms such as pain or anxiety, allowing them to live functional lives and meet daily obligations (Grundmann, 2017; Coe et al., 2019; Smith et al., 2021b).

### Energy and Focus

A longstanding use for kratom in SE Asia is to increase productivity. In a survey of over a million kratom users in this region, a primary motive was to enhance physical performance (Tanguay, 2011). This may explain why a preponderance of traditional consumers are male agricultural laborers. Chewing kratom leaves while working the fields has been embedded in the culture for centuries. A recent analysis of 293 male Malaysian daily consumers revealed their main reason for ingestion was to work longer hours with less fatigue and pain (Singh et al., 2014). In another survey of 136 kratom users (predominantly male) in Malaysia, most reported the motive of increased work capacity and enhanced energy. It appears that Westerners are also increasingly using kratom to improve occupational functioning, much like the common use of coffee. Three Western online surveys (over 16,000 respondents combined) revealed increased energy and improved focus as main reasons for kratom consumption (Grundmann, 2017; Pain News Network, 2017; Coe et al., 2019). Social media analyses of kratom users also found these self-reported motivations and benefits of kratom use (Smith et al., 2021b; Smith KE. et al., 2021). In fact, clinical, scientific, and ethnographic reports spanning from 1930 to 2017 consistently reveal kratom’s role in enhancing, sustaining, or making it feasible to meet work demands (Henningfield et al., 2018).

### Mood and Mental Health

A substantial portion of kratom users report using the plant to improve mood or manage symptoms associated with a mental health diagnosis. In the largest scale survey in SE Asia, many revealed using kratom to “feel better” and “cope with problems” (Tanguay, 2011). In a Western survey of 2,867 current and 157 former kratom users (Coe et al., 2019), 22% reported using kratom to alleviate symptoms of anxiety, post-traumatic stress disorder (PTSD), or depression. In another US-based survey of 8,049 users (Grundmann, 2017), 66% used kratom to treat emotional or mental conditions. Additionally, a survey of 6,150 kratom consumers by Pain News Network (2017) revealed 14.5% of respondents used kratom to treat anxiety, 8.83% to treat depression, and 1.40% to treat insomnia. The most recent survey of kratom users (*n* = 2,798; Garcia-Romeu et al., 2020) indicated that depression and anxiety were the motivation for kratom use for 67% and 65%, respectively. A literature review of 13 peer-reviewed studies in SE Asia and the United States examining kratom use and mental health provided further support that many individuals use kratom as a mood enhancer or anxiolytic (Swogger and Walsh, 2018). Specifically, the stimulant effect at low doses reportedly acts as a mood booster, while higher doses induce relaxation that may alleviate anxiety. There is also preliminary evidence that kratom has empathogenic effects, leading the authors to hypothesize that kratom may enhance sociability beyond what would be accomplished with anxiety reduction alone (Swogger et al., 2015).

### Pain Management

While kratom’s longstanding medicinal use in SE Asia applies to a variety of ailments, alleviation of pain is among the most common. A study in which 562 kratom users were interviewed in Malaysia revealed pain relief as a main reason for consumption (Ahmad and Aziz, 2012). Results of large-scale United States online surveys reveal that pain relief is the most common reason for kratom use (Grundmann, 2017; Pain News Network, 2017; Coe et al., 2019; Garcia-Romeu et al., 2020). The Pain News Network (2017) survey queried pain conditions that people were managing with kratom. The most common identified were back/spine pain, followed by acute pain from injury, fibromyalgia, migraine or headache, and rheumatoid arthritis. Other pain conditions people reported treating with kratom included multiple sclerosis, neuropathy, osteoarthritis, inflammatory bowel disease, lupus or other autoimmune diseases, complex regional pain syndrome, Ehlers-Danlos syndrome, trigeminal neuralgia, and cancer. Over 90% of the respondents indicated that kratom is “very effective” in treating their pain or medical condition, while approximately 7% reported it to be “somewhat effective” (Pain News Network, 2017). Adding to data from observational studies, results from a recent randomized, double-blind, placebo-controlled trial indicated that kratom significantly increased acute pain tolerance, as measured in the laboratory using the cold-pressor task (see Brown et al., 2003), in a sample of 26 male kratom users. (Vicknasingam et al., 2020).

### Harm Reduction

The value of substitution (replacing an undesirable substance with a less harmful one) is evidenced by cannabis as a successful substitute for alcohol, opioids, and cocaine (Bachhuber et al., 2014; Socías et al., 2017) or treating OUD by replacing opioids with high potential for dependence (e.g., heroin, oxycodone) with those with less potential for dependence (e.g., methadone or buprenorphine). Consistent with descriptions of kratom use in SE Asia dating back to the 19th century (Hassan et al., 2013), current research indicates that kratom is being successfully used as a harm-reduction method or self-treatment for opioid withdrawal, including as a short- or long-term opioid substitute (Ward et al., 2011; PinneyAssociates, 2016; Smith and Lawson, 2017; Smith et al., 2021b; Smith KE. et al., 2021). Notably, kratom has the advantage of being available to individuals who cannot access medical treatment due to barriers in the system or will not access it due to mistrust of health care professionals, thus providing a potential self-treatment for OUD to a wide swath of people who would otherwise receive none.

A convenience study sample of 136 kratom users (99% male; mean age = 38.7) in an area of Malaysia known for heavy kratom use revealed that 90% were using kratom as a substitute for opioids and 84% indicated that kratom helped with their opioid withdrawal symptoms (Vicknasingam et al., 2010). In another Malaysian survey (Singh et al., 2015) of 293 adult male regular kratom users (mostly manual laborers; mean age = 28), 15% indicated that they had used kratom in an effort to reduce or eliminate addictions to illicit substances (e.g., opioids, cannabis) and/or to ameliorate opioid withdrawal symptoms. Kratom has also been used in SE Asia as a substitute or self-treatment for amphetamines and alcohol (Vicknasingam, et al., 2010; Singh et al., 2021) and in the United States to self-treat alcohol dependence (Smith et al., 2021b). These self-report data converge with preliminary signals in the pharmacology literature suggesting the therapeutic potential of kratom alkaloids for harmful alcohol use (Gutridge et al., 2019; Gutridge et al., 2020).

In the West, five United States.-based internet surveys of over 20,000 kratom users, as well as over 20,000 comments to the Drug Enforcement Administration (DEA), and a survey of more than 500 people in treatment for opioid use disorder, indicate that many are using kratom as an alternative to opioids (Grundmann, 2017; Smith and Lawson, 2017; Henningfield et al., 2018; Coe et al., 2019; Garcia-Romeu et al., 2020). In one of those surveys (Grundmann, 2017), nearly half of 8,049 respondents indicated that kratom enabled them to reduce or discontinue the use of opioids. Ten percent of 3,017 respondents to another survey (Coe et al., 2019) were taking kratom to cut down on opioid use and/or relieve withdrawal. Of those using kratom in place of opioids, 90% indicated that it was helpful to relieve pain, reduce opioid use, and relieve withdrawal. An analysis of 170 kratom threads during a 12-month period (2004–2005) on a Western online pharmacy indicated that a vast majority purchased kratom to treat opioid withdrawal (Boyer et al., 2007). Furthermore, in a study of 161 respondents to a United States.-based internet forum, over 10% reported using kratom to successfully decrease or abstain from a substance that was unwanted or considered to be causing harm (Swogger et al., 2015). Similar social media analyses indicate that kratom use as an opioid substitute is widespread (Smith et al., 2021b; Smith KE. et al., 2021).

Use of kratom as an effective substitute is supported by preclinical research, which is of particular interest in light of the current United States opioid crisis of rising dependence rates, emergency room visits, and overdose deaths. A recent study by Saref et al. (2019) provided evidence of kratom’s potential as a harm-reduction agent and Yue et al. (2018) found that rodents pre-treated with mitragynine self-administered less heroin. Using a convenience sample of 260 illicit drug users, Saref et al. (2020) found association was found between self-reported initiation of kratom and reduction in both the consumption of various illicit drugs and frequency of HIV risk behaviors related to sexual practice and injection drug use. These findings are promising, given that estimated calculations put morphine-like opioids at an overdose risk of *a thousand or more times* that of kratom (Henningfield et al., 2019).

Kratom as a substitute to opioids also has the potential to improve social, family and occupational outcomes and behavior (Swogger et al., 2015; Henningfield et al., 2018; Swogger and Walsh, 2018). Like coffee drinkers, regular kratom users often consume this herbal supplement as a beverage in the company of others, enhancing social connection. In contrast, long-term daily opioid use can lead to self-isolation since this drug, unlike kratom, is conducive to the quick intense euphoria attained through snorting, injecting, and inhaling (Henningfield et al., 2018). In fact, only 2% of 6,135 kratom-users in the Pain News Network (2017) online survey responded “yes” to the question “Can you get high from kratom?” In addition to survey data, thousands of public comments to DEA and FDA attested to the successful substitution of kratom for opioids (Prozialeck et al., 2019).

### Adverse Kratom Effects and Kratom Withdrawal

Despite centuries of kratom use in SE Asia, there have been few reports of serious adverse events associated with its use, and kratom overdose has not been identified as a direct cause of death in fatalities coincident with kratom use (PinneyAssociates, 2016). Among a sample of 293 SE Asian dependent kratom users, none reported having to obtain medical treatment related to kratom use (Singh et al., 2014). Recent research in the West confirms that adverse effects appear to be rare and dose dependent. In Grundmann’s (2017) survey of 8,049 kratom users, less than 1% sought medical or mental health treatment related to kratom consumption, similar to low rates of adverse effects or healthcare treatment utilization for kratom found by Garcia-Romeu et al. Dosages of at least 5 g and frequency of 22 or more times per week were more likely to be associated with side effects (occurring in approximately 20% of 3,024 respondents), which were primarily gastrointestinal in nature (nausea, constipation, etc.). Other reported side effects of kratom use include vomiting, drowsiness, irritability, agitation, headache, runny nose, watery eyes, weight loss, insomnia, dehydration, and excessive thirst (Vicknasingam et al., 2010; Adkins et al., 2011; Anwar et al., 2016; Lydecker et al., 2016; Singh et al., 2016; Grundmann, 2017). These predominantly self-managed side effects occurred in about 13% of 3,024 respondents in the Coe et al. (2019) survey.

Kratom tolerance, dependence, and withdrawal have been reported with daily and heavy use, though these symptoms are generally milder and of shorter duration than those of classical opioids (Ahmad and Aziz, 2012; Singh et al., 2014; Singh et al., 2015; Swogger et al., 2015; Grundmann, 2017; Swogger and Walsh, 2018; Smith et al., 2021b). Physical dependence that can develop over time has been described as similar to that of coffee or mild opioid dependence (Brown et al., 2017). A study on dependent users (three or more daily servings) indicated that withdrawal symptoms (including insomnia, nausea, vomiting, diarrhea, muscle pain or spasms, shakiness, runny eyes or nose, and hot flashes) resolved within one to 3 days for most (Singh et al., 2014). Longer duration of use and higher average dose may extend the duration and increase the severity of withdrawal, however, and a small number of individuals may find kratom very difficult to quit (Smith et al., 2021b).

Overall, there appears to be minimal, short-term risk to the majority of people using kratom with the intention of self-treating a variety of conditions. While these findings warrant validation in controlled clinical studies, they reveal that for many people, kratom enhances their health in ways they report as unachievable, or with fewer side effects, than by other means, including pharmaceuticals. For this reason, it is important that healthcare practitioners are prepared and willing to have conversations with their patients about the use of kratom products pending greater scientific understanding of this plant and experimental validation of its traditional and (growing) conventional uses.

## Best Practices in the Clinical Care of People Who Use Kratom

Up to 60% of patients turn to non-medical modalities for treatment (Alwhaibi et al., 2015) and over 30% use herbal-based remedies, especially for conditions involving chronic pain (Barnes et al., 2008; Pain News Network, 2017). Although the majority of kratom consumers do not reveal their use to healthcare providers (Grundmann, 2017; Coe et al., 2019), popular use of natural remedies is evident on “pharmacy watch” websites, such as *drugs.com,* that disseminate information regarding alternatives for pain management, including kratom (Boyer et al., 2007). Healthcare practitioners must therefore be knowledgeable about the implications of using these substances. Indeed, the National Institute on Drug Abuse (NIDA) advises that “people should check with their healthcare providers about the safety of mixing kratom with other medicines” (NIDA, 2019), suggesting that patients do turn to their providers for information. Meanwhile, providers and patients alike have been put in unnecessarily difficult positions as they attempt to sort out contradictions between United States government agency-fueled headlines (e.g., the CDC warning that kratom may cause psychosis or death; Anwar et al., 2016) and more reasonable interpretations of the existing scientific data on kratom. To aid in this process, and with the earlier stated caveats that kratom has not been approved as safe and effective for any medical disorder and we are not encouraging or endorsing such use, we offer *best practices* for assessing and treating people who use kratom, pending further study in controlled experiments.

### Assessment

It is important to contextualize kratom assessment for the patient in a way that feels consistent with the non-judgmental and routine nature of a competent medical or mental health evaluation. Embedding questions about the use of “herbal medicines, like Valerian root or kratom” in an assessment of pharmaceuticals and supplements acknowledges kratom’s place among other treatments that people choose to use. The stance is non-stigmatizing and respectful and may increase the likelihood of honest patient disclosure. Initiating a discussion with open-ended questions about patients’ experiences with kratom, desired outcomes, and concerns enables practitioners to assess gaps in knowledge or false beliefs, areas for patient education. Moreover, apprehending patients’ motivations for use also enables clinicians to provide education around other, FDA-approved treatments such as cognitive-behavioral therapy for anxiety and buprenorphine for opioid replacement. Kratom does not appear in standard drug screens (White, 2018). Regardless, we do not recommend routine screening for illicit substances without consideration of the potential impact on patient-provider trust, which may be necessary for honesty and positive outcomes.

### Patient-Centered Conceptualization

It is helpful for providers to explicitly state that they are unable to recommend or condone the use of kratom or any substance that is not approved by the FDA, but that they can provide education and work to understand the patient’s kratom use. The non-judgmental approach to assessment recommended above leads naturally to a conceptualization of kratom use within the context of individual patient values and goals. The evidence-based treatment *process* specifically highlights the need for clinical engagement and shared decision-making with patient values and goals inherent in that process (Gambrill, 2006; Melnyk et al., 2010; Hoffmann et al., 2014). Treatment around substance use that involves patient values has evidence for efficacy (Osaji et al., 2020). Someone who, for example, is unsuccessfully using kratom to self-treat anxiety may be surprised to learn that there are evidence-based psychotherapies (e.g., cognitive-behavioral therapy) that are effective for doing the same. Someone who is self-treating OUD may wish to switch to buprenorphine, or other medication-assisted therapy if they are unhappy with their response to kratom or wish to use an FDA-approved product. For people without histories of opioid dependence, however, switching from kratom to buprenorphine or methadone should be considered judiciously on a patient-by-patient basis. Assessment of patient values and goals can lead to rich, meaningful discussions of the potential risks and benefits of a number of treatments and can contribute to trust, and thus effectiveness, in the clinician-provider relationship.

### Understand Patient Motives for Use

With the explicit acknowledgment that data from randomized controlled trials is lacking, the available data reviewed above indicate that people across the world consistently report that kratom is useful for increasing energy, improving mood and alleviating anxiety, and for decreasing and/or ceasing opioid use and alleviating related withdrawal symptoms. Some people have reported that kratom has been a helpful substitute for other substances that are causing harm (e.g., alcohol). Finally, the observational literature indicates that people consistently report the utility of kratom for pain relief, and this is corroborated by the only clinical trial to examine kratom and analgesia in humans.

### Dosing

The literature on dosing is consistent, but imprecise. In general, lower doses up to approximately 5 g of raw plant material are reported to exert stimulant effects and have been compared to caffeine. Doses between approximately 5 and 15 g are reported to lead to relaxation and analgesic effects. These higher doses may be necessary for successful opioid substitution. Side effects are more likely at higher doses. Limited data show that there is variability in terms of lowest and highest perceived dose as being ineffective or effective, and that feelings of discomfort may arise from higher doses (Smith et al.). Although no dose or dosing range can be clinically recommended, but patients can be informed that some average doses are approximately 2.5 g (Smith et al.). The development of kratom dependence, including tolerance and withdrawal symptoms upon cessation or reduction, is more likely at higher doses and with frequent, recurring use. For this reason, patients should be encouraged to use as little as needed for therapeutic effects. People who have decided to initiate kratom use should begin with a minute amount to test for adverse reactions before slowly increasing. It is important to convey that the potency of the plant can vary based on factors such as geographical source, the season, age of the sample, and post-harvest handling (Adkins et al., 2011; Griffin and Webb, 2018; Pearson et al., 2018; Zhang et al., 2020), as well as strain, which is commonly referred to as vein type (red, green, or white) and likely corresponds to the age of the leaf. Evidence suggests that the red vein variety may be more potent than the older, green vein (Braley and Hondrogiannis, 2020). Patients should be advised to purchase kratom from the same manufacturer to be as consistent as possible in the product (see further discussion below).

### Duration of Action

Generally, the effects of kratom last for approximately five to 7 hours, with the strongest effect within two to 4 hours of ingestion, though aftereffects (e.g., fatigue) can be felt as late as the following day (Maruyama et al., 2009; Prozialeck et al., 2012; Rosenbaum et al., 2012; Scott et al., 2014). One should not assume, however, that effects of kratom will not “kick in” sooner, especially if taken on an empty stomach, or last longer due to individual variation.

### Side Effects and Adverse Effects

Unwanted effects associated with kratom use have been systematically studied. Large scale surveys and other observational studies of tens of thousands of users reveal that a minority experience dose-dependent side effects that are mostly mild and self-resolve. The most common of these are constipation, nausea, vomiting, other stomach irritation, and drowsiness (Grundmann, 2017; Swogger and Walsh, 2018). Dizziness and sedation are possible, so patients should avoid driving or any other activities one would avoid after drinking alcohol. Adverse effects, while rare, cannot be ruled out. These include the potential for liver problems, seizures, and dependence (discussed below). Individuals with compromised liver, kidney, or cardiac function may be at increased risk for harm (Harizal et al., 2010). While a recent study found no QTc interval prolongation differences between kratom users and controls (Leong Abdullah et al., 2021), it is worth noting that a small number of cases allude to this possibility and cardiotoxicity in humans cannot be ruled out (see Lu et al., 2014). Clinical trials may reveal additional long-term and/or rare side effects and adverse effects.

### Kratom-Drug Interactions

As discussed earlier, it is important to note the possibility of risky drug interactions between kratom and alcohol, opioids, and benzodiazepines for instance, which may induce respiratory depression, as well as use in combination with stimulants because kratom also as stimulant effects. In the absence of evidence of more detailed safety data, there should be the presumption of some level of risk of kratom use in combination with other pharmacologically active substances and that such risks are more likely to be increased by higher levels of consumption. Thus, patients taking kratom should be made aware that interaction effects with other substances have not been studied. It is therefore highly advisable to refrain from mixing kratom with other substances including herbal products. Additionally, mitragynine’s observed mechanisms of action can guide warnings issued to kratom consumers taking certain medications. The alkaloid’s stimulation of postsynaptic alpha-2 adrenergic receptors (Prozialeck et al., 2012) suggests that it can accentuate and therefore be dangerous with other sedative, hypnotic, and analgesic drugs (White, 2018). Additionally, a recent *in vitro* study revealed the possibility that kratom ingestion mixed with drugs that are P-gp substrates (e.g., erythromycin, loperamide, protease inhibitors) could lead to clinically significant toxicity (Rusli et al., 2019). Finally, there is evidence that kratom cytochrome P450 enzyme activities, thus raising the possibility of herb-drug interactions when administered along with agents that use the same metabolic pathway (Hanapi et al., 2013).

### Dependence

Kratom dependence occurs for a minority of users, especially at high doses with frequent dosing. Individuals with substance dependence histories who are considering initiating kratom use should be informed that their risk of dependence is theoretically higher than for people who have never had harmful substance use. Solid therapeutic relationships enable patients to be honest about their kratom use, allowing for close monitoring of increased signs of dependence (e.g., tolerance, cravings, and withdrawal symptoms upon cessation). Patients looking to decrease or discontinue kratom use can be assured that this is often done without medical intervention (Saingam et al., 2016) and that a gradual taper is recommended. Mild withdrawal effects occur in some people and usually resolve within days. These may include physical symptoms (muscle spasms or soreness, diarrhea, muscle aches, lack of appetite, fever, and runny eyes and nose) and psychological symptoms (mood swings, irritability, nervousness restlessness, disturbed sleep, tension and sadness; Singh et al., 2014). Some people who have ceased use report substituting coffee or energy drinks to help with withdrawal symptoms (Saingam et al., 2016). Patients should be encouraged to call their provider if concerns arise regarding the severity or duration of their withdrawal symptoms, as more severe and long-lasting withdrawal has been reported. In such cases, initiation of an opioid substitute (e.g., buprenorphine) and/or supportive psychotherapy may be beneficial. Patients who experience more severe withdrawal symptoms should be cautioned against making important decisions until they are feeling better and should be encouraged to contact their provider if they begin having thoughts of suicide due to mood changes occurring during the withdrawal process.

### Additional Considerations

In order to purchase kratom that is of consistent potency and is unadulterated, patients should be informed that vendors who use Good Manufacturing Practices (GMP) are identifiable. It is also potentially helpful to inform patients about whether they risk arrest for use of kratom in a particular jurisdiction (see Table 1). Finally, it may be helpful to clarify that FDA non-approval of kratom as a treatment, despite the significant observational evidence for utility, reflects the fact that clinical trials to substantiate users’ reported effects have yet to be conducted. In conclusion, we present here the most up-to-date information regarding kratom to inform healthcare providers with the necessary data to have honest and straightforward discussions with their patients concerning kratom use, which is currently on the rise in the United States, and could have important health ramifications.

**TABLE 1:** Legality of kratom in United States.

State	Legality
Alabama	Illegal in all areas for use, possession, and purchase
Arkansas	
Indiana	
Vermont	
Wisconsin	
Rhode Island	
Illinois	Legal for use, with exception of Jerseyville, Alton, and Edwardsville, to people over the age of 18
New Hampshire	Legal to use for individuals over the age of 18, except for Franklin City
California	Legal for use but banned in the city of San Diego
Florida	Legal for use but banned in Sarasota County
Mississippi	Banned in 33 counties and towns but remains legal in the rest of the State
Colorado	Legal in Colorado, with exceptions in Parker and Monument towns. Denver is illegal for human consumption
Tennessee	Legal to sell as long as it’s labeled and in its natural botanical form (Pure)**.** Legal to use for individuals over the age of 21
Arizona	Kratom Consumer Protection Act passed and enacted, kratom products need to follow GMP manufacturing guidelines and labeling standards set by the state legislature
Georgia	
Nevada	
Utah	
Remaining states	Legal to consume, purchase and sell. In many cases, you must be an adult over 18
